# Lymphocytic Colitis Is Associated with Increased Pro-Inflammatory Cytokine Profile and Up Regulation of Prostaglandin Receptor EP4

**DOI:** 10.1371/journal.pone.0061891

**Published:** 2013-04-17

**Authors:** Indranil Dey, Paul L. Beck, Kris Chadee

**Affiliations:** 1 Departments of Microbiology, Immunology and Infectious Disease, Health Sciences Centre, Snyder Institute for Chronic Inflammation, University of Calgary, Calgary, Alberta, Canada; 2 Division of Gastroenterology, Health Sciences Centre, Snyder Institute for Chronic Inflammation, University of Calgary, Calgary, Alberta, Canada; INSERM, France

## Abstract

Microscopic colitis (MC) is comprised of two entities, lymphocytic (LC) and collagenous colitis. Up to 20% of patients with chronic diarrhea that have a normal appearing colonoscopy will be diagnosed with MC. Since MC was first described less than 40 years ago, little is known about the mechanisms involved in disease pathogenesis. Nonsteroidal anti-inflammatory drugs are associated with an increased risk of MC and some reports suggest a dysregulation in prostaglandin production. Recent genome wide screens have found an association between prostaglandin receptor EP4 expression and inflammatory bowel disease; however, EP4 expression has never been studied in MC. The aim of this study was to assess colonic mucosal inflammatory cytokine profiles in patients with LC and to assess expression of the prostaglandin receptor EP4. Colonic mucosal biopsies were obtained from patients undergoing colonoscopy for investigation of diarrhea and in those undergoing colon cancer screening. Following histological assessment, expression of cytokines and the prostaglandin receptor EP4 was analyzed using real-time reverse transcriptase-PCR and immunohistochemistry. Patients with LC had markedly increased mRNA expression for TNF-α, IFN-γ and IL-8 compared to normal controls (p<0.001). No significant differences were noted for IL-1β, IL-4, IL-10 or IL-12/23. Interestingly, those with LC had increased EP4 receptor expression, which positively correlated with increased TNF-α expression. This is the first report to demonstrate that LC is associated with increased TNF-α, INF-γ and IL-8 concurrent with a marked up-regulation of EP4. These findings add to our knowledge on the pathogenesis of LC and may give rise to possible new therapeutic and/or diagnostic tools in the management of MC.

## Introduction

Microscopic colitis (MC) is common cause of chronic diarrhea in patients having a normal mucosa upon colonoscopy. Although once considered rare, recent studies [1], [2] have shown that the incidence is increasing and in some regions the incidence is similar to that of Crohn's disease. MC includes two main entities: collagenous colitis (CC), lymphocytic colitis (LC) [3]. The histopathology of CC is characterized by sub epithelial collagen deposition and LC is characterized by a marked intraepithelial lymphocytosis [4]. The estimated incidence of MC has been estimated to be 4.2–10.0 per 100,000 [1], [5]. We have recently reported [1] on one of the largest cohort of MC patients to date and found an annual incidence rate of 10.0 per 100,000 person-years (lymphocytic colitis, 5.4; collagenous colitis, 4.6 per 100,000). The main risk factors associated with the development of MC includes; female gender, age (marked increased risk after age 60 yr), presence of other autoimmune diseases [1], [6]–[8], smoking [9], [10], solid organ transplant recipients [11] and certain drugs [12]. Although many drugs have been associated with an increased risk of MC, non-steroidal anti-inflammatory drugs (NSAIDs) have been the most commonly reported [12], [13]. Several studies report that those with MC are more likely to have been exposed to NSAIDs and that withdrawal of NSAIDs may improve disease severity [14]–[16].

Although the cytokine profiles of Crohn's disease (Th1, Th17/23) and ulcerative colitis (predominantly Th2) have been well studied, there is little known about the mediators that may be involved in the pathogenesis of MC. To our knowledge, there are only few studies that have been published on the inflammatory cytokines in MC [17]–[19]. Current studies suggest that MC patients might be genetically predisposed to the disease. One study identified a positive correlation between IL-6-174 gene polymorphism and MC [17]. Another study showed that both CC and LC were associated with the HLA-DR3-DQ2 haplotype (18). Previous studies [19] suggest that patients with MC predominantly had a Th1 type cytokine profile with marked increases in interferon gamma (IFN-γ), and interleukin (IL) 15, tumor necrosis factor alpha (TNF-α) an increased inducible nitric oxide synthase (iNOS) while other study [20] found increased levels of TGF-β in patients with CC.

The role of prostaglandins and their receptors in MC is unknown. One case report [21] showed that a patient with CC had extremely high luminal excretion of PGE_2_ compared to a control subject and they implicated these high PG levels in the profuse fluid and electrolyte secretion in this patient. Apart from that single study, there are no studies on PG's, PG receptors or COX-1/2 in MC. One could hypothesize that prostaglandin levels would be increased in MC since prostaglandins and COX-2 are increased in Crohn's disease and ulcerative colitis [22]–[24]. Furthermore, several of the above inflammatory mediators, including TNF-α, INF-γ, IL-1, that are increased in MC can up regulate COX-2 mediated PGE_2_ production [25]–[26]. PGE_2_-EP receptor interactions may have a critical role in homeostasis or in the onset of GI inflammation [27]. EP receptors play a major role in exerting pro/anti-inflammatory effects of PGE_2_. However, limited data suggest that signaling via different EP receptors can predetermine whether PGE_2_ exerts a pro-inflammatory or anti-inflammatory effect. Signaling through EP_2_ and EP_4_ receptors (via Gs subunit) leads to increased levels of intracellular cAMP an important second messenger for gene regulation and other cellular functions [27]–[29]. The main roles that have been described for prostaglandin in the gastrointestinal tract include enhancing barrier function, wound repair and restitution following injury, regulation of mucosal blood flow and mucus production/secretion. PGs also play numerous roles in immune regulation. Our studies [29]–[30] have shown that PGE_2_ exerts pro-inflammatory responses by up-regulating IL-8 in colonic epithelial cells. Another study found that PGE_2_ exacerbates intestinal inflammation via signaling through EP4 receptors in dendritic cells resulting in up regulation of the pro-inflammatory cytokines IL-17 and IL-23 [31]. PGs can regulate T cell responses of the mucosal immune system and one study revealed that EP4 receptor expression on CD3+ve T cells is increased with intestinal inflammation. This study also showed that PGE_2_-EP_4_ signaling in intestinal T cells resulted in a decrease in inflammatory cytokine production [32]. The involvement of EP4 in intestinal inflammation is also supported by studies that found targeted deletion of EP4 resulted in increased susceptibility to colitis in a murine model system [33] and a specific EP4 agonist reduced the severity of colitis [34]. More recently, a genome wide screen found an association between Crohn's disease and genetic variants in the gene coding for EP4 receptors [35]. Thus, it appears that EP receptor-PG signaling can have both pro- and anti-inflammatory action in gastrointestinal mucosa. With the hypothesis that dysregulation of events involved in prostaglandin signaling may play a critical role in the pathogenesis of MC, the aims of this study was to assess colonic mucosal cytokine expression and expression of the EP2 and EP4 receptor subtypes.

## Materials and Methods

### Tissue Collection

Colonic biopsies were obtained from patients undergoing colonoscopy for the investigation of chronic diarrhea or for colon cancer screening. Samples were placed in RNA-later (Qiagen) and stored at −80°C until further processing. Some tissues were also fixed in formalin and processed in a standard fashion for analysis by our pathology department. The Department of pathology reviewed all the biopsy specimens and normal histology was confirmed in the control subjects (colon cancer screening patients). The diagnostic criteria for LC were: 1) intraepithelial lymphocytosis (≥20 IEL per 100 surface epithelial cells); 2) inflammation in the lamina propria consisting of mainly lymphocytes and plasma cells; 3) epithelial damage, such as flattening and detachment and 4) sub-epithelial collagen layer not present or less than <10 µm. Informed consent was obtained from all patients and the study was approved by the by the Calgary Health Region Conjoint Health Research Ethics Board. Eight patients (5 females, 3 males, average age 61) with LC and 12 controls (7 females, 5 males, average age 59.6) were included in the study. None of the patients were taking on NSAIDs, aspirin or other anti-inflammatory medications within 7 days of the colonoscopy.

### RNA isolation and quantitation of gene expression

Biopsies were obtained during colonoscopy and immediately rinsed in PBS and then placed in RNA Later (Ambio, Austin, Texas) and frozen at −20°C. RNA was isolated from the biopsy samples with Trizol (Invitrogen Life Technologies, Burlington, ON) as per manufacture's protocol. Reverse transcription of total RNA was performed using a commercial kit, 1st Strand cDNA Synthesis Kit for reverse transcriptase (RT)-PCR (Invitrogen), according to the manufacturer's instructions. The resulting complementary DNA (cDNA) was diluted 1 in 10 and the samples stored at −20°C until processing by PCR.

Quantitative real-time RT-PCR was performed using the Lightcycler instrument (Rotor Gene from Corbett Life Science), Qiagen's Quantitect SYBR Green PCR kit and the following primers, TNF-α: TCAGTCAGTGGCCCAGAAGAC (forward), GATACCCC-TCACACTCCCCAT (reverse); IFNγ: ACATTCCACAATTGATTTTATTCTTACAA-CA (forward), ACGAGCTTTAAAAGATAGTTCCAAACA (reverse); IL-10: CTCCCC-TTACCTTCTACACACAC (forward), CCTTACTTTCCACTTACCTATCCC (reverse); IL-4: ACGACCTGTCCTTCTCAAAACACTA (forward), AGAGGCAGAATAACA-GGCAGACTCT (reverse); IL-1β: ATTGCTCAAGTGTCTGAAGC (forward), GTAGT-GGTGGTCGGAGATT (reverse); IL-12/23 (12-p40 subunit which is shared by both IL-12 and IL-23): CTGCAGTTAGGTTCTGATCCAG (forward), GGCAACTTGAGAGC-TGGAAA (reverse). IL-8: CGTGGCTCTCTTGGCAGC (forward), TCTTTAGCACT-CCTTGGCAAAAC (reverse); EP_2_: ATGGGCAATGCCTCCAATG (forward), TCAA-AGGTCAGCCAGTTTAC (reverse); EP_4_: ACGCCGCCTACTCCTACATG (forward), AGAGGACGGTGGCGAGAAT (reverse); GAPDH: GAAGGTGAAGGTCGGAGT (forward), GAAGATGGTGATGGGATTTC (reverse). Primers were designed to amplify messenger but not genomic DNA. The relative quantitation method was undertaken using the Lightcycler relative quantitation software (Rotor Gene). Positive controls (cDNA provided in the kit) and negative controls (no cDNA) were included in all experiments, and RT-PCR results were validated by melting curve analysis. All experiments were performed in duplicate according to the manufacturer's instructions, with final results presented as an average of duplicate values. Expression of mRNA was normalized against GAPDH and fold change over control was determined according to Ct method as previously described [29].

### Immunohistochemistry

Paraffin-embedded sections were deparaffinized and placed in Target Unmasking Fluid (Cedarlane, Hornby, ON) to enhance antigen retrieval. Sections were washed in 0.1 M TRIS–HCl/0.005% bovine serum albumin (BSA) and incubated overnight at 4°C with either EP4 antibody (Cayman Chemical, Ann Arbor, MI) or an isotype control antibody (Normal Rabbit IgG, R&D Systems, Minneapolis, MN). Antibodies were diluted in 0.1 M TRIS–HCl/0.005% BSA/10% normal goat serum (NGS). After three washes in 0.1 M TRIS–HCl, sections were incubated in secondary antibody (anti-mouse CY3, 1∶1,000; Jackson Laboratories, West Grove, PA.) for 1 h at room temperature. Immunoreactivity was assessed by standard microscopy in a blinded fashion.

### Statistical analysis

Data are presented as mean ± standard error of the mean (SEM). When required data were analyzed by two-way ANOVA and Student's t-test for unpaired data using Graphpad Prism software 4.0. Significance was considered at p≤0.05.

## Results

### Cytokine mRNA analysis

**Figure 1:**
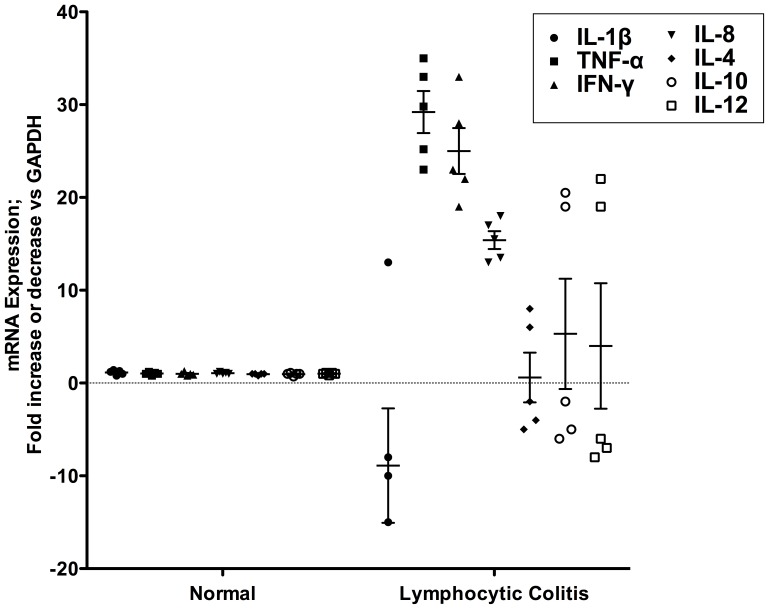
Cytokine mRNA expression profiles from normal controls and lymphocytic colitis patients. Total RNA was extracted from biopsy samples and RT-PCR was performed using primers specific for different cytokines as described in material and methods. The value for each patient shown in the scatter plot represents fold change of cytokine mRNAs expression over normal controls patients repeated three times. Mean values for each data set is shown by the horizontal bar ± standard error of the mean. *** p<0.001 for TNF-α, IFN-γ and IL-8 versus normal controls.

As there is no definitive diagnostic test for IBD, we compared the expression profiles of several Th1 and Th2 type cytokines in LC patients by RT-PCR (Fig. 1). We hypothesize that dysregulation of cytokine production may play a major role in the maintenance and or propagation of chronic inflammation in the gut. As shown in Figure 1, the pro-inflammatory cytokines TNF-α, IFN-γ and IL-8 mRNAs were significantly (p<0.001) over expressed (over 10-fold more) in the biopsy samples of LC patients compared to normal controls. Interestingly, IL-1β, which is normally over expressed in IBD, remained consistently low. An interesting finding was that the regulatory cytokines IL-4, IL-10 and IL-12 did not significantly increase above basal values (Figure 1).

### Differential expression of EP4 receptor

**Figure 2:**
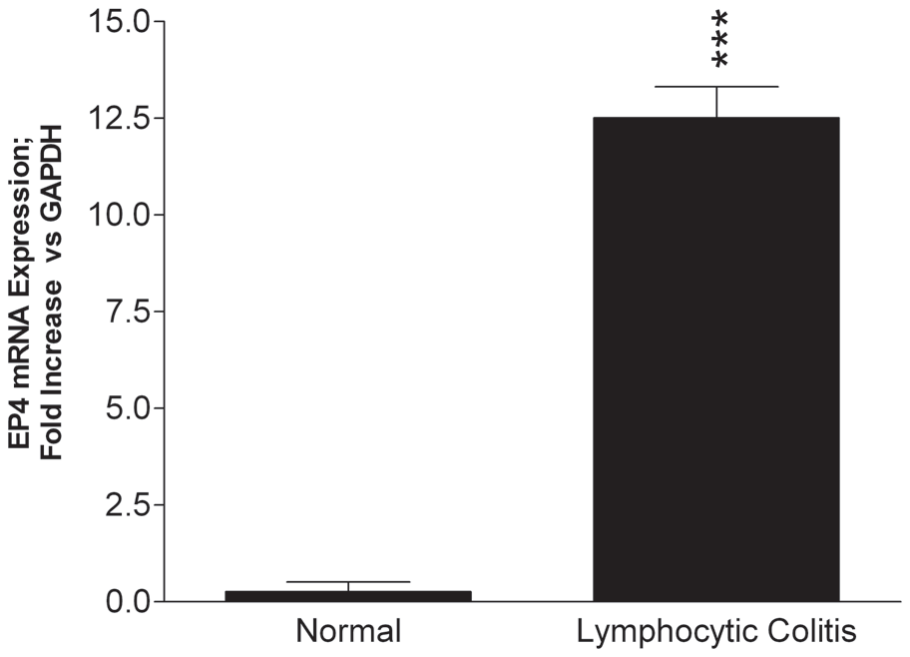
EP4 receptor mRNA expression in LC patients. Note there was significant up regulation of EP4 receptor mRNA levels in LC patients as compared to controls, *** p<0.001.

**Figure 3:**
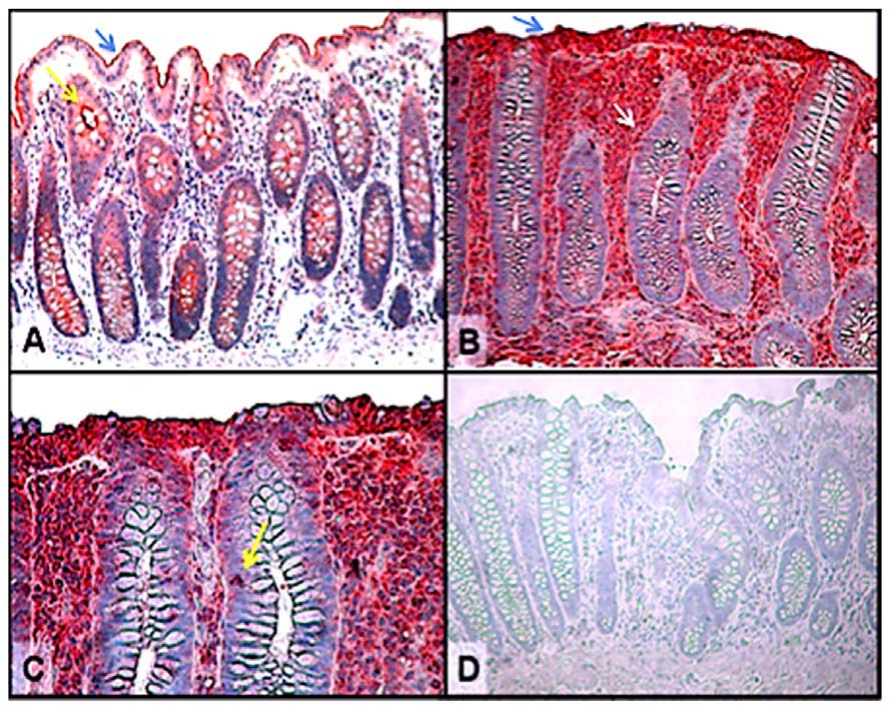
Immunohistochemical localization of the EP4 receptor in lymphocytic colitis tissues. A. Normal control colon demonstrating EP4 staining (blue arrow) localized to the surface of intestinal and crypt (yellow arrow) epithelial cells. B and C. Lymphocytic colitis tissues showing increased EP4 expression on surface intestinal epithelial cells (blue arrow) as well as the infiltrating lymphocytes within the epithelium (yellow arrow) and lamina propria (white arrow). D. Control tissues exposed to isotype primary antibody demonstrating specificity for EP4 staining. Original magnification (x 400).

**Figure 4:**
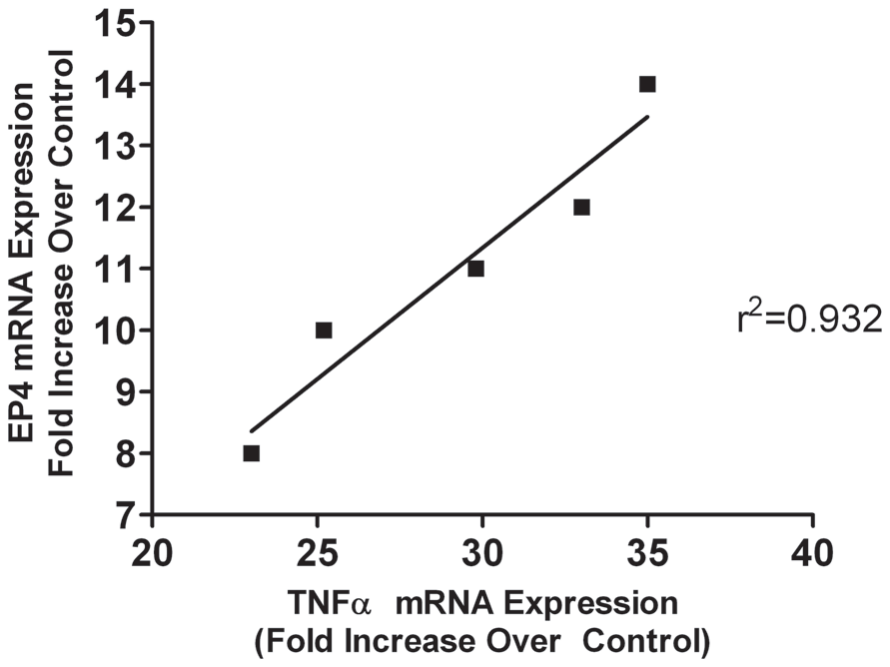
Correlation between TNF-α mRNA expression and EP4 mRNA expression in patients with lymphocytic colitis. Note, no significant correlation was noted in normal control patients nor with other cytokines assessed in Figure 1.

Arachidonic acid metabolites and in particular, PGE_2_ are increasingly being implicated in the pathogenesis of inflammatory bowel disease. It is well established that the expression of EP-receptors, especially those of the EP4 subtype are markedly altered during colonic inflammation and their expression and signaling play a major pro-inflammatory role in the pathogenesis of colitis [22], [27]. As shown in Figure 2, in LC patients the expression profiles of EP4 mRNA were significantly (p<0.001) up regulated as compared to normal controls. EP2 mRNA was also modestly elevated but not as robust at the EP4 receptor subtype (data not shown). These results were corroborated by immunohistochemistry data demonstrating the presence of increased EP4 expression in intestinal epithelial cells as well as in infiltrating lymphocytes within the epithelium and lamina propria (Fig. 3). Remarkably, our data indicate that there was a strong correlation between elevated levels of TNF-α expression and increase EP4 receptor mRNA expression in patients with LC (Fig. 4). Changes in EP4 receptor mRNA expression was highly correlated (r^2^ = 0.932) with TNF-α mRNA expression. Somewhat surprising is that even though IFN-γ and IL-8 mRNA were significantly up regulated in LC patients (Fig. 1), EP receptor expression was not. Similarly, EP4 receptor expression did not significantly correlate with changes in IL-1β, IL-4, IL-10 or IL-12 mRNAs expression in LC patients.

## Discussion

Since both LC and CC are relatively recently described entities, numerous critical aspects of these disease states are still emerging. Although MC is associated with autoimmune diseases and has been linked to some medications including NSAIDs and aspirin, little is known about the pathogenesis of these disease states. Even the natural history of MC is unclear. Clearly, it is more common in females and the risk of MC increases with age [1]. We and others [1], [3], [36]–[38] have reported that MC is an increasingly common cause of chronic diarrhea especially in those over the age of 60 and thus, we can expect further increases in incidence as our population ages. Although MC is a common cause of chronic diarrhea, little is known about either the inflammatory response or the mechanisms involved in the pathogenesis of these disease states. The present study clearly shows that LC is associated with increased in pro-inflammatory cytokines and that there is an increase in the expression of prostaglandin receptor EP4, which may be involved in increased fluid excretion and inflammation associated with LC. Recent studies [39] showed that serotonin might also play an important role in lymphocytic colitis by interacting between immune and serotonin cells.

IL-1β, IFN-γ and TNF-α have also been shown to induce a number of other changes in epithelial cells that allow them to function as accessory immune cells. Furthermore, TNF- α and IFN-γ have recently been shown to be important in the promotion of an efficient innate immune response by intestinal epithelial cells [40]–[41]. The high levels of TNF-α and IFN-γ association with patients with CD and MC as compared with controls is reminiscent of that described in mucosa of patients with celiac disease [22]. IFN-γ and TNF-α seem to be key cytokines in the gut that causes non-specific tissue damage [41]. Our findings of a marked increase in TNF-α, IFN-γ and IL-8 expression in MC is consistent with a pro-inflammatory state involving the up regulation of the innate immune system. This is likely to be a key event in signaling between the intestinal epithelium and the innate immune system, leading to the attraction and activation of immune cells. IFN-γ and TNF-α have a critical effect on intestinal permeability. *In vitro* studies using human intestinal epithelial cell monolayers have demonstrated a reversible decrease in tight junction permeability [42]. Increased paracellular permeability is likely to be a key component in the induction of diarrhea during IBD and MC [43]. It is well documented that inflamed and non-inflamed intestinal tissues in UC and CD have impaired and permeable mucosal barriers [44], [45].

We have recently discussed the diverse role that PGE_2_-EP receptor signaling pathway plays in normal homeostasis and in gastrointestinal inflammation [27]. PGE_2_ levels are significantly increased during inflammatory bowel disease but its role in the pathogenesis of the disease and in particularly, in MC remain undefined. There is very limited data available on the role of PGE_2_ and different EP receptors in gastrointestinal inflammation and MC. The actions of PGE_2_ are mediated by G-protein-coupled receptors EP1-EP4 [27], [29]. EP_2_- and EP_4_-receptors couples via G_s_, to adenylyl cyclase to produce intracellular cAMP. PGE_2_ has both pro/anti-inflammatory properties in the gut and signaling via particular EP receptors can predetermine whether PGE_2_ exerts a pro-inflammatory or anti-inflammatory effect. However, despite this commonality in signaling, stimulation of EP_2_- and EP_4_-receptors can evoke different responses. For example, in DSS induced colitis, EP_4_ receptor agonist suppresses of colitis induced damage by up regulating IL-10 [46] or through increased mucus secretion [47]. In contrast, we have shown [29], [30] that PGE_2_ up-regulates IL-8 production in colonic epithelial cells than can cause non-specific tissue damage by chemo-attracting/activating neutrophils. More recently [48] we showed the mechanism whereby EP4 receptor and prostaglandin transporters play in PGE_2_-induced alteration of epithelial barrier integrity in rodent colitis and patients with IBD. Moreover, PGE_2_ acting via EP2/4-receptor inhibits cytokine productions in macrophages [33], [34]. A recent report [35] showed that EP_4_-receptors were up regulated in the colon of colorectal cancer patients and that an EP_4_-receptor antagonist suppressed carcinogenesis.

In conclusion, this study demonstrates that the mucosal cytokine profile in LC is of a Th1 phenotype, with features closest to those previously documented for celiac disease. Many of the morphological features noted in LC are shared with celiac disease that may be explained by the cytokine profile. EP-receptors are altered during different stages of intestinal inflammation and our studies reinforces that elevated EP4 receptor mRNA expression correlated with high levels of TNF-α could represent an ideal biomarkers for LC.
